# *In Vivo* Confocal Microscopic Observations of Vortex Keratopathy in Patients with Amiodarone-Induced Keratopathy and Fabry Disease

**DOI:** 10.1155/2018/5315137

**Published:** 2018-03-21

**Authors:** Yasuhito Ikegawa, Atsushi Shiraishi, Yasuhito Hayashi, Akiyoshi Ogimoto, Yuichi Ohashi

**Affiliations:** ^1^Department of Ophthalmology, Ehime University Graduate School of Medicine, Shitsukawa, Toon, Ehime 791-0295, Japan; ^2^Department of Cardiology, Ehime University Graduate School of Medicine, Shitsukawa, Toon, Ehime 791-0295, Japan

## Abstract

**Purpose:**

To compare the morphology of two types of vortex keratopathy: amiodarone-induced keratopathy and the Fabry disease-associated keratopathy.

**Patients and Methods:**

Eight patients who were receiving oral amiodarone therapy and 3 patients with Fabry disease, a mother and her 2 daughters, were examined by slit-lamp biomicroscopy and *in vivo* confocal microscopy (IVCM) regularly.

**Results:**

Amiodarone-induced keratopathy developed in 7 of the 8 patients, and it was detected as early as 7 days by IVCM and 14 days by slit-lamp biomicroscopy. The *in vivo* confocal microscopic images showed a clustering of corneal epithelial cells with a highly reflective cytoplasm in both types of keratopathy. In the amiodarone-induced keratopathy, the highly reflective epithelial cells were first found at the center of the cornea and then spread to the periphery with increasing time on amiodarone. In Fabry disease, the highly reflective epithelial cells were consistently observed extending from the limbus to the central cornea.

**Conclusion:**

These findings suggest that the corneal epithelial cells most likely endocytose amiodarone from the tear film in the amiodarone-induced keratopathy. In Fabry disease, globotriaosylceramide deposits are taken up by the lysosomes of the limbal epithelial stem cells, and they differentiate and migrate to the center of the cornea to form the whorl pattern.

## 1. Introduction

A whorl-shaped corneal dystrophy is present in patients with Fabry disease named vortex keratopathy or cornea verticillata because of the whorl-shaped appearance of the cornea [1]. Fabry disease is caused by a genetic mutation at q22 of the X chromosome, and it results in a deficiency of the alpha-galactosidase A enzyme. The incidence of Fabry disease is 1 in 40,000 to 117,000 individuals in European studies [2]. In Fabry disease, there is a progressive increase in the deposition of globotriaosylceramide (Gb3), a sphingolipid degradation product, within the lysosomes of the epithelial cells of various organs. The deposits lead to various side effects, for example, hypohidrosis, cardiomyopathy, renal failure, and vortex keratopathy [2, 3]. The vortex keratopathy is manifested by opacities that swirl from a point below the center of the cornea and radiate to the peripheral cornea. The keratopathy is typically bilateral and initially asymptomatic and can be seen as early as 6 months of age [1–6]. There are also less common ocular findings in Fabry disease including cataracts and conjunctival and retinal vessel tortuosity [3, 6, 7].

Amiodarone is a class III antiarrhythmic drug that is effective for the treatment of various types of tachyarrhythmias. It penetrates lysosomes and binds to cellular lipids producing a drug-induced lipidosis [8]. Amiodarone has several side effects including thyroid dysfunction, pulmonary fibrosis, neuropathy, gastrointestinal problems, and drug interactions [9]. The ocular side effects of amiodarone have been reported in the optic nerve, retina, lens, eyelids, and cornea [10–12]. Among these, amiodarone-induced keratopathy is the most common ocular side effect, and it is characterized by bilateral, golden-brown, whorl-shaped deposits in the cornea. Amiodarone-induced keratopathy has been reported to develop 1 to 3 months after beginning the amiodarone and is progressive. However, it does not affect the vision. After the discontinuation of amiodarone, the keratopathy improves and is resolved in 3 to 20 months [11, 13, 14].

Recent advances in *in vivo* confocal microscopy (IVCM) have allowed clinicians to examine the corneal morphology in greater detail. The morphological changes of vortex keratopathy in Fabry disease and amiodarone-induced keratopathy have been examined by IVCM [8, 15–19]. Among these, two studies compared the two types of corneal keratopathy; Falke et al. reported that the hyperreflective deposits were present in the basal cell layer of the corneal epithelium in both types of diseases, and Wasielica-Poslednik et al. reported different microstructural changes in the two types of cornea keratopathy [17, 20]. Thus, it was established that the hyperreflective deposits were in the corneal epithelial cells in both Fabry diseases and amiodarone-induced keratopathy. However, a more precise morphological description of these two diseases is still needed.

Thus, the purpose of this study was to compare the morphological appearance and time course of the development of the cornea keratopathy in amiodarone-induced keratopathy to that in Fabry disease. The morphological characteristics detected by slit-lamp biomicroscopy were compared to those detected by IVCM. In addition, we attempted to determine the pathogenesis of these two keratopathies, and the clinical course of amiodarone-induced keratopathy was examined regularly by slit-lamp biomicroscopy and IVCM.

## 2. Materials and Methods

### 2.1. Subjects

Sixteen eyes of eight patients (5 men and 3 women) with a mean ± standard deviation age of 73.9 ± 11.9 years were studied. Oral amiodarone was prescribed to 8 patients, and they were examined by slit-lamp biomicroscopy and IVCM periodically from the beginning of the amiodarone treatment. The Heidelberg Retina Tomograph II-Rostock Cornea Module (HRT II-RCM) (Heidelberg Engineering, Heidelberg, Germany) was used to obtain the IVCM images. The amiodarone dosage ranged from 50 to 200 mg/day. The subjects were examined before and 1, 2, and 4 weeks and 2, 4, 6, and 12 months after beginning the amiodarone therapy. The duration and dosage of the drugs were recorded until the detection of amiodarone-induced keratopathy, and the final grading score of the keratopathy was done according to that used by Orlando et al. [21].

A mother and two daughters, ages 32, 8, and 4 years, respectively, who were diagnosed with Fabry disease, were examined by slit-lamp biomicroscopy and IVCM. The younger daughter was examined only by slit-lamp biomicroscopy and not by IVCM due to her young age.

The procedures used in this study were approved by the Institutional Review Board of Ehime University, number 1502001. An informed consent for the injections, examinations, and measurements was obtained from all the subjects, and the procedures used conformed to the tenets of the Declaration of Helsinki.

## 3. Results

### 3.1. Amiodarone-Induced Keratopathy

**Figure 1:**
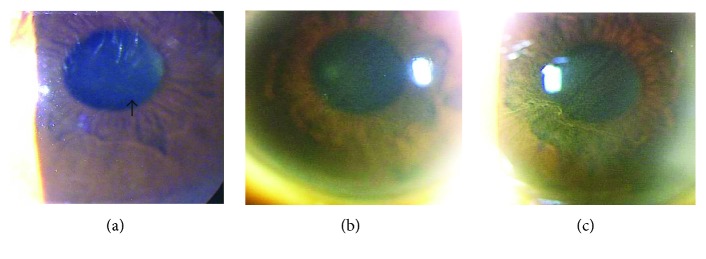
Slit-lamp biomicroscopic findings of amiodarone-induced keratopathy of the right eye of case 1. (a) Photograph of the anterior segment of the eye 39 days after amiodarone application. A line of white deposits can be seen just inferior to the center of the cornea (arrow). (b) Photograph of the anterior segment of the eye 128 days after amiodarone application. More arborizing horizontal lines can be seen just below the center of the cornea. (c) Photograph of the anterior segment of the eye 194 days after amiodarone application. The arborizing lines have a whorl-like pattern extending from the visual axis toward the periphery.

Vortex keratopathy was detected by slit-lamp biomicroscopy and IVCM in 7 of the 8 patients who were being treated with oral amiodarone, that is, amiodarone-induced keratopathy. The keratopathy was first detected as horizontal linear deposits located just inferior to the center of the cornea. With time, additional arborizing horizontal lines developed and extended to form a whorl pattern (Figure 1). The amiodarone-induced keratopathy was first detected by IVCM at an average of 32.0 ± 24.8 days and by slit-lamp biomicroscopy at an average interval of 67.4 ± 59.8 days after beginning amiodarone therapy. The difference in the intervals was not significant (*p* = 0.08; Table 1).

The average dose of the drugs at the time when the amiodarone-induced keratopathy was first observed was 4178.6 ± 1773.9 mg by IVCM and 10,950 ± 12367.7 mg by slit-lamp biomicroscopy. This difference was not significant (*p* = 0.17; Table 1).

**Figure 2:**
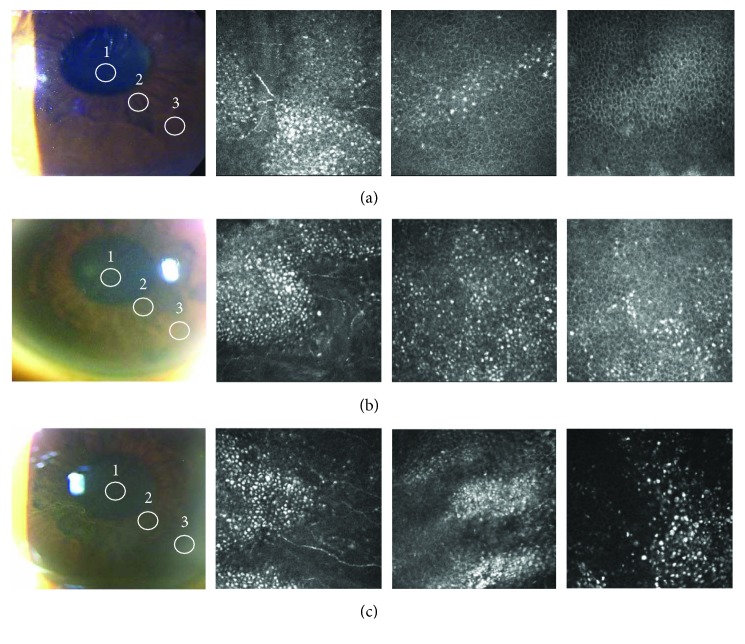
*In vivo* confocal microscopic findings of the right eye of case 1 at 39 days (a), 128 days (b), and 194 days (c) after beginning the amiodarone application. The corneal basal epithelial cell layers from the center (1), paracentral (2), and peripheral (3) cornea (circles) are shown. (a, 1) Clusters of epithelial cells with highly reflective cytoplasm can be seen at the center of cornea. (a, 2) Sparsely clustered highly reflective epithelial cells can be seen at the paracentral cornea. (a, 3) No highly reflective epithelial cells are observed at the peripheral cornea. (b, 1; b, 2) Clusters of epithelial cells with a highly reflective cytoplasm can be seen at the center (b, 1) and paracentral (b, 2) cornea, and the clusters are often seen aligned. (b, 3) Sparsely clustered highly reflective epithelial cells are observed at the peripheral cornea. (c) Clusters of epithelial cells with a highly reflective cytoplasm can be seen at the center (c, 1), paracentral (c, 2) and peripheral (c, 3) cornea, and the clusters are often aligned.

**Figure 3:**
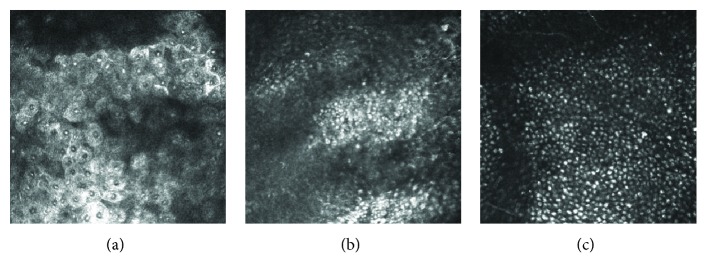
*In vivo* confocal microscopic findings of superficial (a), suprabasal (b), and basal (c) cell layers of the right eye of case 1. Highly reflective epithelial cells can be seen in the basal layer to the superficial layers of the corneal epithelial cells.

At the beginning of the amiodarone-induced keratopathy, IVCM showed clusters of epithelial cells with a highly reflective cytoplasm at the center of the cornea and loosely clustered, highly reflective epithelial cells also in the paracentral epithelial cells (Figure 2). However, the highly reflective epithelial cells were not observed in the peripheral corneal epithelial cells (Figure 2). As the keratopathy progressed, clusters of epithelial cells with highly reflective cytoplasm were found in the peripheral epithelial cells (Figure 2), and they were observed in the basal to the superficial layers (Figure 3).

The *in vivo* confocal microscopy revealed microdots in the stroma in 5 of 8 patients and in the endothelial cells in 2 patients with amiodarone-induced keratopathy. The average time for the microdots to appear was 53.2 ± 18.7 days in the stroma (Table 1). The microdots were detected in the stroma 25.2 days after the appearance of the highly reflective epithelial cells (Table 1). The time for the microdots to be detected in the endothelium was 32 and 63 days after beginning the amiodarone, and they were 0 and 28 days after the microdots appeared in the stroma in 2 patients (Table 1).

### 3.2. Fabry Disease

**Figure 4:**
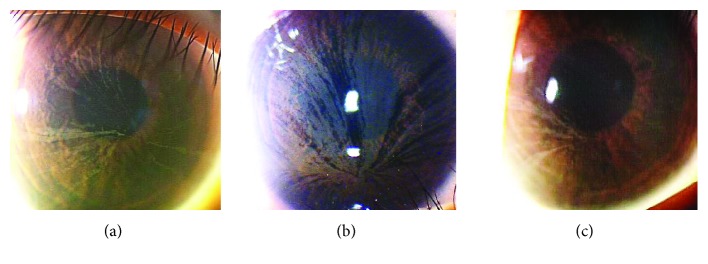
Slit-lamp biomicroscopic findings of three patients with Fabry disease. (a) Findings in the 32-year-old mother. (b) Findings in the 8-year-old daughter. (c) Findings in the 4-year-old daughter.

**Figure 5:**
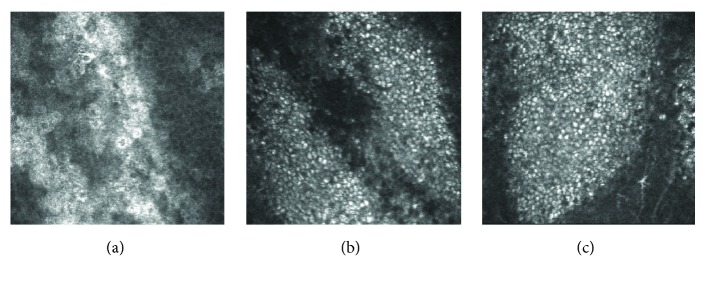
*In vivo* confocal microscopic findings of the superficial (a), suprabasal (b), and basal (c) epithelial layers in the right eye of the 32-year-old mother with Fabry desease. The highly reflective epithelial cells can be seen in the basal layer to the superficial corneal epithelial cell layers.

**Figure 6:**
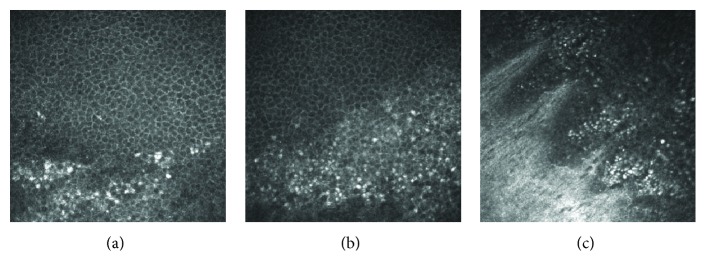
*In vivo* confocal microscopic findings of the right eye of the 32-year-old mother with Fabry disease. The corneal basal epithelial cell layer is seen at the center (a), paracentral (b), and limbal (c) regions of the cornea. Clusters of epithelial cells with a highly reflective cytoplasm can be seen at the center, paracentral, and limbal regions of the cornea, and the clusters are often seen aligned.

In the 3 patients with Fabry disease, slit-lamp biomicroscopy detected white or yellow powdery corneal deposits arranged in a whorl pattern (Figure 4). The *in vivo* confocal microscopical examinations revealed that clusters of cells with a highly reflective cytoplasm were present in the superficial to the basal layers of the corneal epithelium (Figure 5). These patterns resembled those detected in the amiodarone patients. In contrast to the amiodarone-induced keratopathy, the highly reflective epithelial cells were consistently observed extending from the corneal limbus to the central cornea in Fabry disease (Figure 6). Microdots were not found in the stroma and endothelium in the two Fabry patients by IVCM. The third 4-year-old daughter was examined only by slit-lamp biomicroscopy, and her findings were similar to those of her mother and older sister.

## 4. Discussion

The *in vivo* confocal microscopic examinations showed highly reflective corneal epithelial cells in 7 of the 8 amiodarone patients and in 2 of the patients with Fabry disease. The *in vivo* confocal microscopic examinations also showed microdots in the stroma in 5 and in the endothelium in 2 of the 8 amiodarone patients but in none of the Fabry disease patients. The amiodaron-induced keratopathy was detected as early as 7 days after beginning the amiodarone by IVCM and 14 days by slit-lamp biomicroscopy. These findings are in good agreement with previous reports [8, 15–19]. The pathological findings were detected earlier in 4 of 7 amiodarone patients by IVCM which may be because IVCM can detect cellular changes which cannot be detected by slit-lamp biomicroscopy, although the difference in the detection time was not statistically significant.

Amiodarone has high affinity for lipids in the cells especially in the lysosomes, and their interactions result in the accumulation of intracytoplasmic lamellar inclusion bodies. These inclusion bodies have been found in the lung, heart, liver, adipose tissue, and cornea [9]. In Fabry disease, deficient activity of the enzyme, alpha-galactosidase A, results in the deposits of sphingolipid degradation Gb3 in lysosomes of various organs including the cornea [2, 5]. Thus, the highly reflective epithelial cells detected by IVCM may indicate the presence of deposits of amiodarone or Gb3 in the lysosomes of the epithelial cells in these two keratopathies. We were not able to differentiate any morphological differences of the highly reflective epithelial cells in the two conditions by the IVCM findings. This may be because of the small number of cases; however, the limited black and white resolution of the current IVCM device may not be able to obtain an accurate morphological appearance of the cytoplasm of the corneal epithelial cells. It may also be possible that different doses of amiodarone or different enzyme levels in Fabry disease might affect the cytoplasmic deposits which are the cause of the high reflectivity of the cytoplasm.

An interesting finding of this study was the changes in the pattern of the highly reflective epithelial cells during the clinical course in the amiodarone patients. At the beginning, the highly reflective epithelial cells were observed only in the central area of the cornea, and as the amiodarone-induced keratopathy progressed, the highly reflective epithelial cells spread from the center to the peripheral cornea. However, the highly reflective epithelial cells were not observed at the limbus even when the amiodarone-induced keratopathy was advanced. These findings suggest that the corneal epithelial cells endocytose amiodarone from the tear film during their centripetal migration. This is possible because it has been reported that amiodarone is present in the tear fluids [22]. The idea that the corneal epithelial cells endocytose amiodarone from the tear film is supported by the observation of microdots in the stroma and endothelial cells after being detected in epithelial cells because it requires a longer time for the tear fluids to penetrate through the stroma and endothelium compared to the epithelium. Previous reports and our results showed that it requires about 1 to 3 months to detect amiodarone keratopathy [11, 13, 14] indicating that it may take time for the endocytosed amiodarone to be detected by IVCM as the highly reflective material by the corneal epithelial cells.

In contrast, highly reflective epithelial cells were consistently observed to be present at the limbus to the central cornea in Fabry disease. This suggests that the deposition of Gb3 within the lysosomes is occurring in the limbal epithelial stem cells, and the limbal epithelial stem cells with the Gb3 deposits differentiate and migrate toward the center of the cornea.

It is generally accepted that the corneal epithelial stem cells are in the basal epithelial layer at the limbus, and they differentiate and migrate centripetally across the cornea to the central area [23–26]. This pattern of migration was recently tested in genetically modified mice, and the pattern of the visualized corneal epithelial cells in the experimental animals was quite similar to that of the cells in the eyes with vortex keratopathy associated with amiodarone and Fabry disease [27–29]. Thus, the slit-lamp biomicroscopic observations in Fabry disease along with the IVCM findings of groups of highly reflective epithelial cells observed to line from the limbus to the central cornea indicate that these cells may be of the same lineage as the limbal epithelial stem cells [27, 30]. Although the limbal epithelial cells were not positive in the amiodarone-induced keratopathy, they were similar in appearance by IVCM and slit-lamp biomicroscopy at the peripheral to the central cornea. These findings may be because they have the same lineage as the corneal epithelial cells that have differentiated from one limbal epithelial stem cell and have the same affinity to uptake amiodarone as the highly reflective epithelial cells.

There are some limitations in our study. First, the limited resolution of the model of IVCM used did not allow us to differentiate the cytoplasmic changes in the two diseases in more detail. Second, our hypothesis was based on the different patterns of the highly reflective epithelial cells between the two diseases and during the clinical course of the amiodarone-induced keratopathy. However, strong conclusions cannot be made because of the small number of patients, and studies with a larger number of patients are needed to be able to understand the morphological mechanisms of the two diseases.

This prospective observational study by IVCM allowed us to follow the pathological changes during the clinical course *in vivo* and helped to test our hypothesis. This study recognized that repeatable and less invasive observation by IVCM can be a useful method to examine corneal diseases.

In conclusion, we found different patterns of the highly reflective corneal epithelial cells in the amiodarone-induced keratopathy and Fabry disease by IVCM. According to the different patterns, we hypothesized that the corneal epithelial cells endocytose amiodarone from the tear film during the centripetal migration. In Fabry disease, the deposits of Gb3 within lysosomes occur in the limbal epithelial stem cells, and the cells differentiate and migrate to the center of the cornea.

## Figures and Tables

**Table 1:** Clinical Findings of Amiodarone Patients.

			Durations and dosage of drugs until vortex keratopathy was detected				Durations until microdots were detected by IVCM		Final stage of vortex keratopathy	
			By IVCM		By slit-lamp microscopy					
Case number	Sex	Age	Duration (days)	Dosage (mg)	Duration (days)	Dosage (mg)	Stroma (days)	Endothelium (days)	Stage OD/OS	Days OD/OS
1	M	48	7	2600	39	9000	74	ND	3/3	194/194
2	W	82	35	3600	63	6400	35	63	3/3	190/190
3	M	72	14	3200	14	3200	60	ND	3/3	181/181
4	M	81	67	6700	192	38,600	ND	ND	2/2	374/374
5	M	70	21	4400	21	4400	ND	ND	2/2	173/173
6	W	77	15	2300	78	8600	32	32	2/2	183/183
7	W	87	65	6450	65	6450	65	ND	1/1	339/339
8	M	74	ND	ND	ND	ND	ND	ND	0/0	180/180
Average		73.9	32.0	4178.6	67.4	10,950	53.2	47.5	2/2	226.8/226.8
